# Parenting Programme Structure, Potential Barriers, and Facilitators: A Scoping Review

**DOI:** 10.3390/ijerph192013655

**Published:** 2022-10-21

**Authors:** Ana Ramos, Filomena Matos, Hélia Soares

**Affiliations:** 1 Instituto Politécnico de Setúbal, Escola Superior de Saúde. NURSE’IN-UIESI, Campus do IPS, Estefanilha, 2910-761 Setúbal, Portugal; 2 University of Algarve, Health School. UICISA: E. NURSE’IN-UIESI, University of Algarve, Campus de Gambelas, 8005-139 Faro, Portugal; 3 University of Azores, Health School. NURSE’IN-UIESI, University of Azores, 9700-042 Angra do Heroísmo, Portugal

**Keywords:** parental stress, parenting education, child development, family health, parenting, scoping review

## Abstract

Becoming a parent is a challenging transition, and stress factors may arise. This scoping review aims to map, from the literature, the structure, potential barriers, and facilitators to be considered when conducting a parenting programme for parents of children up to 3 years old. It followed the JBI methodology and included studies with parents of children up to 3 years old (Participants), studies about parenting programme structure, its potential barriers, and facilitators (Concept) in the healthcare or community setting (Context). Qualitative and/or quantitative study designs and grey literature publications between 2016 and 2021 were eligible. The search was performed in three stages in CINAHL Plus with Full Text, MEDLINE with Full Text, and PubMed databases. It was also conducted in OpenGrey, ProQuest, Portuguese Open Access Scientific Repository, and Google Scholar. Fourteen articles were selected. The following aspects were identified regarding parenting programmes: benefits; structuring elements to be considered; facilitating factors and possible barriers to its development, and measurement instruments to assess the programme. Parenting programmes are important ways to contribute to a healthy, sustainable, and resilient society. It should be adapted to individuals, groups, and communities. They add value to parents, children, and society and should be carefully adapted to the group’s needs.

## 1. Introduction

Healthy development during childhood promotes vigorous adulthood, contributing to a productive and sustainable society. A family household is where children establish first relationships, attachment, and continuous interaction, learn to trust and become acquainted with basic social rules [1]. However, it is also where most child abuse occurs, often caused by a parent [2,3,4,5,6]. This toxic stress impact on the early years triggers adverse adulthood outcomes and shortens life expectancy [7,8,9,10].

Different kinds of stress can be induced: positive, tolerable, and toxic. The first is a normal part of healthy functioning; the second is more intense and long-lasting but can be buffered by supportive relations allowing the nervous system to get back on track; toxic stress arises from prolonged adverse childhood experiences (ACEs) such as reiterated abuse, chronic neglect, exposure to violence, or accumulated economic hardship in the family [8].

Parents and family are those closest to the child and are expected to be the best caregivers [11]. However, cumulative toxic stress can occur without appropriate parental support, which may cause development disruption [12] and promote morbidity before adulthood and early death [8,12].

The COVID-19 pandemic has put children and caregivers at even higher risk by promoting inequalities with long-term impacts [12,13]. Studies show that this abrupt change in daily life harms the individual’s mental health and increases depression and anxiety [13]. Chronic stress, due to adversity, lays a foundation for adverse outcomes in both physical and mental health since it generates an inadequate response of inflammation from the nervous system that affects the metabolic and immune systems deeply [7,8,12] without proper backing.

Early childhood development (ECD) science explains that the factors that influence health outcomes and the achievement of development potential depend not only on biological or behavioural characteristics but also on the experience and environment in which the child develops [14,15,16].

Additionally, the 4.2 Goal Target of the United Nations Sustainable Development Goal is “to ensure that all girls and boys have access to quality early childhood development, care, and pre-primary education” [17]. However, the current policy landscape has not yet resulted in the implementation of large-scale national parenting programmes. Thus, more scientific research is needed to adapt and deliver parenting programmes in diverse local contexts, coordinate within existing systems and services, fund programmes to be cost-effective and sustainable, and scale-up parenting programmes while maintaining quality and effectiveness [18].

Effective parenting is critical in fostering healthy child development [19] and responsive care, which is the caregiver’s ability to understand the child’s needs and respond appropriately, promoting growth and buffering stress response. However, for some parents, a social support network, parental education and support programmes are essential. Thus, parental education and support programmes that strengthen informal social support networks are needed [20].

This study supports its framework in three theoretical models, which are complementary and interconnected: the child-centred care model, the family-centred care model and the nurturing care framework.

The child-centred care model places the children and their interests as the care focus [21]. The parenting programmes to be developed must have this purpose, considering they seek to support the family in promoting the child’s well-being.

Naturally, that parenting programmes aimed at parents of children up to 3 years old need to be based on a family-centred care philosophy approach that respects the specificity of each family, informs them, increases their literacy, and helps to support the decisions that best suit their needs and contexts [22].

The Nurturing Care Framework “provides strategic directions for supporting the holistic development of children from pregnancy up to age 3” [15]. This emphasises the importance of early childhood development and the components of nurturing care that should be present in parenting programmes: good health, adequate nutrition, responsive caregiving, opportunities for early learning and security and safety.

The brain is particularly receptive to experience during the early development phase. Investing in this period is one of the best ways to eliminate inequality, boost shared prosperity and create human capital for economic growth [15,23].

This article is focused on parenting programmes for parents of children up to three years old. The choice of the age group up to 3 years is related to early childhood development and the opportunity that the first years of life must influence the child’s growth and development. On the other hand, it corresponds to the period in which children spend more time with their families, highlighting the importance of this interaction.

A preliminary search of MEDLINE, the Cochrane Database of Systematic Reviews, JBI Evidence Synthesis, PubMed, and PROSPERO, was conducted. No current systematic or scoping reviews on all the specific questions were identified. 

Thus, this scoping review was registered in OSF Registries (OSF Registries|Parenting education training structure, potential barriers and facilitators factors: a scoping review protocol). The scoping review was developed using the JBI Scoping Review framework [24], the PRISMA-Scoping Review extension [25], and the guide of Pollock et al. [26].

This scoping review aimed to map in the literature the aspects to be considered when structuring a parenting programme for parents of children up to 3 years of age. The review question was: What information is available in the literature about parenting programme structure, potential barriers, and facilitators for parents of children up to 3 years old in a healthcare setting or community?

More specifically, this scoping review aims to answer the following questions:

- What are the benefits of parenting programmes?
- What aspects should be considered in structuring a parenting programme for parents of children up to 3 years?
- What facilitating aspects were found in the literature?
- What are the potential barriers that were found in the literature?
- What measurement tools and indicators can be monitored to evaluate parenting programmes?

## 2. Materials and Methods

Using the participants, concept, and context (PCC) framework [24], the scoping review included studies that: (a) participants were parents of children up to 3 years old; (b) focused on the concept of parenting programme structure, its potential barriers, and facilitators; (c) occurred in the context of healthcare setting or community. 

The inclusion criteria were: (1) studies in English and Portuguese, with full text available, (2) studies published between 2016 and 2021, (3) studies with qualitative and/or quantitative methodology and grey literature, (4) studies with parents of children up to 3 years old, (5) completed studies with results.

The time limit is aimed at collecting the most current evidence, given the dynamics of contemporary society and parenthood. Studies with parents of children up to 3 years were included, not excluding studies with a different age range, provided that children up to 3 years of age were integrated. This option aims to gather more information about the existing programmes in the literature and allows integration of our study’s target population: the parents of children up to 3 years old.

The exclusion criteria were: (1) study protocols, (2) studies on educational interventions for infants in neonatal intensive care units with children with specific disorders, diseases, or conditions; (3) studies about programmes aimed at adolescent parents and parents in situations of psychosocial vulnerability.

This review aims to identify the aspects to be considered in a universal parenting programme. Programmes aimed at parents in specific situations were excluded because those families’ needs may differ from those of other families.

The search strategy followed that advocated by the Joanna Briggs Institute [24] and Pollock et al. [26]. It included published and unpublished studies and was conducted in three stages: (1) Limited initial search in MEDLINE with Full Text (via EBSCO), CINAHL Plus with Full Text (via EBSCO), and PubMed databases, followed by an identification of text words in the title and abstract of retrieved papers, and of the index terms used to describe the articles; (2) Second search using the search terms identified, in all databases included (Table 1); (3) the third search conducted in the references of all articles and reports identified from the initial search.

A broad search strategy was used to cover the issues in the review mentioned above. 

As advocated by the Joanna Briggs Institute [24], data were extracted from papers included in the scoping review by two independent reviewers. Data were extracted into a table created by the authors, adapted from Pollock et al. [26], including the author(s), year of publication, origin/country of origin, population, context, concept, methodology, and outcomes. If necessary, a third reviewer was ready to resolve reviewer disagreements [24]. There were no conflicts between the evaluators when selecting and extracting the data. 

**Figure 1:**
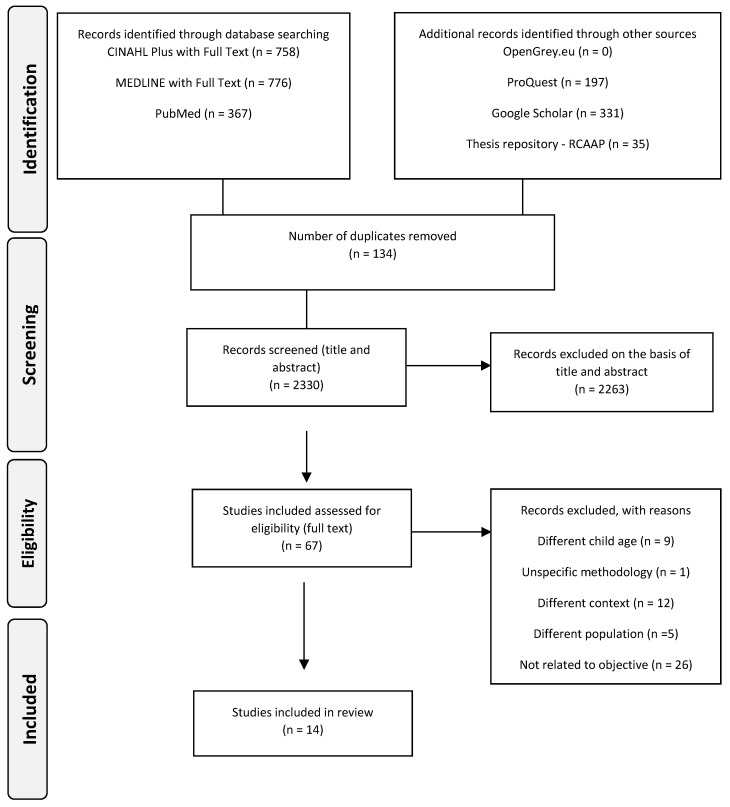
Preferred Reporting Items for Systematic and Meta-Analyses (PRISMA), adapted for scoping review, Flow Diagram.

Figure 1 illustrates the selection process, according to the Preferred Reporting Items for Systematic and Meta-Analyses (PRISMA), adapted for scoping review, Flow Diagram.

As can be seen, 2330 scientific articles were identified after removing those duplicates. Of these, 2263 were excluded after reading the title and abstract and applying the inclusion and exclusion criteria, leaving 67 articles eligible for a full reading.

After full reading, 14 articles were included in this scoping review after independent analysis by two reviewers. The reasons for exclusion are explained in Figure 1. There were no conflicts in data selection and extraction, so there was no need for a third reviewer.

## 3. Results

The origins of the 14 studies were diverse, although studies from Australia (n = 4), the United States of America (n = 3), and China (n = 3) predominated.

The design of the included studies was a randomised controlled trial (n = 6), mixed method (n = 5), qualitative (n = 2), and quantitative (n = 1).

Although the Critical Appraisal and quality of the articles are not mandatory in scoping reviews, according to the JBI, this aspect was considered. For this purpose, the research databases were carefully selected. The articles included in the scoping review are integrated, according to SCImago, in the first quartile (eleven studies), second quartile (two studies) and third quartile (one study) journals, which corroborates the rigour in the selection of studies. In addition, the selected articles were analysed considering the critical appraisal tools made available by the JBI. Only the studies with more than 75% of positive items were included.

The data extracted are presented in Table 2.

### 3.1. Benefits of Parenting Programmes

According to the studies included in this scoping review, the benefits of parenting programmes are evident for parents, the parent and child relationship, and society as a whole.

Parenting programmes can increase parenting competence, skills and practices [27,31,38], self-efficacy [37] and parents’ knowledge about children’s temperament and developmental stages [28,38]. It also enabled the strengthening of social support [31,37] and the way to solve children’s conduct problems [36,38]. The demonstration of various feelings of stress and frustration concerning day-to-day parenting was also reported as one of the benefits of the parental programme [29].

Moreover, the results report that parenting programmes can reduce stress [27,39] and depression [27,37].

Parents reported that the parenting programme relieved societal pressure on parents, allowing them to focus on their relationships with their children. Parents also referred that parenting programmes made it easier for them to understand their children as individuals with their interests and feelings [27] and contributed to child–parent behavioural changes [28].

The results suggest that parenting programmes can have benefits that transcend the family nucleus. Parents shared concepts, strategies, and attitudes among themselves, family members, friends, teachers, and day-care providers [30]. The results of this scoping review indicate other benefits of parenting programmes, such as the better utilisation of child health care resources [19] and a reduction in unplanned consultations and readmissions [40].

### 3.2. Structure of a Parenting Programme for Parents of Children up to 3 Years of Age

The studies suggest that motivational interviewing may be essential in promoting participants’ engagement efforts in parenting interventions [32] and the coherence between the contents and methodology toward target parents’ needs and conditions [33]. A structured curriculum in a programme geared towards providing play and social opportunities was associated with better enrolment than the more typical structured parent group [33].

Factors that contributed to positive experiences included: access for future reference and content more specific to the child’s age and more sortable [30], consistency and continuity between programme modules, using concrete examples to demonstrate concepts, supportive facilitators and group companions, providing childcare, using concrete examples to illustrate parenting concepts, and flexibility in programme delivery [38].

### 3.3. Facilitators of Parenting Programmes

Including children in the sessions was mentioned in the studies as a facilitating aspect [27]. Another facilitating aspect reported by parents was the existence of weekly challenges set, adapted to each one’s reality, to practice the strategies learned [27].

The use of digital technologies and information in digital format was another aspect that parents considered facilitating the parenting programme [27,30]. Accessible content using pictures to promote understanding of the content by less literate mothers [28] and focused on strategies that parents can experiment [29] were also identified as facilitator factors.

Parents reported that the reward system integrated into the programme was an essential motivator for participation [30]. Studies indicate that providing an in-home coach could help vulnerable parents identify and overcome practical barriers [33]. The existence of group facilitators [33] and peer support groups [29] contributed to supporting parents’ active participation.

### 3.4. Potential Barriers to a Parenting Programme

Two main themes emerged concerning barriers to autonomy-supporting parenting practices: (1) lack of empowerment to influence children’s preferences; and (2) stress, fatigue, or lack of time can make parenting challenging. Parents indicated that they had difficulty applying this knowledge. They wanted guidance on translating their knowledge into effective strategies [29].

Time lost was the most common barrier [30]. Parents stated that the many demands placed on them throughout the day made it difficult to “cope” or handle challenges as they arose, particularly at the end of the day. Parents also expressed not having enough time or being too tired to use supportive parenting practices [29]. Suppose the parenting programme is planned to be face-to-face. In that case, the distance between families and parenting centres is essential to be aware of because geographical distance remains a significant barrier to participation in the programme [34].

Technical problems hindering participation have also been reported, such as the requirement of work email addresses during registration, and insufficient internet network, among others [30].

### 3.5. Measurement Tools and Indicators to Monitor and Evaluate Parenting Programme

According to the studies in the scoping review, several dimensions can be evaluated in a parenting programme. In addition to assessing satisfaction with the programme, other indicators can be considered, such as parenting skills, parental stress, social support, depression, parenting sense of competence, and self-efficacy.

**Figure 2:**
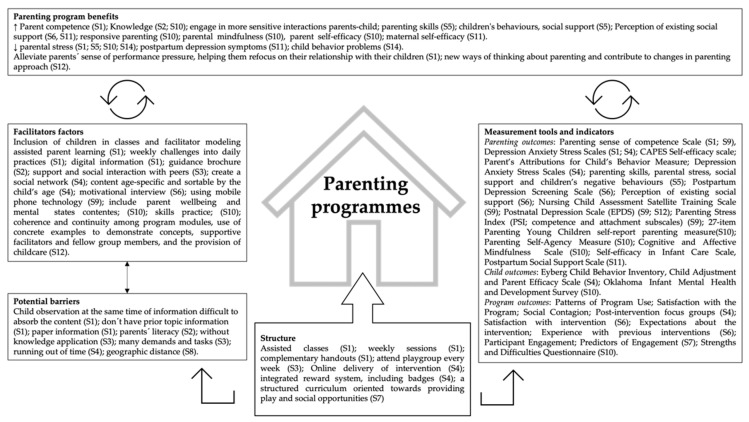
Key findings: benefits, structure, facilitator factors, potential barriers, measurement tools, and indicators of Parenting Programmes.

In addition to the narrative description, a visual summary of the results is presented in Figure 2.

## 4. Discussion

This scoping review aimed to map in the literature the benefits of a parenting programme and the aspects to be considered when structuring a parenting programme for parents of children up to 3 years of age. 

Several parenting programmes were found in the literature that have been implemented worldwide.

This article brings together in a single document the aspects that professionals should consider when implementing a successful parenting programme.

The evaluation of previous experiences will allow a better adjustment to the real needs of the populations and how these parenting programmes can help families to develop their role better, promoting their well-being, as well as the well-being of children, with positive repercussions for society.

Promoting tailored early guidance programmes to families’ strengths and needs [41] improves effective engagement and outcomes for families by increasing parenting quality [42,43]. A couple-centred psycho-educational programme can successfully promote father involvement in parenting [44].

Studies mention virtual delivery as a facilitating factor for parenting programmes, reaching more parents and expanding their networks. Other studies referred to facilitating elements that corroborate the results of this scoping review: a structure according to each family’s needs [45], considering parents’ disposition [42] and reducing distances and costs [46].

Cultural barriers cannot also be forgotten [45]; thus, any materials produced should be culturally relevant and meet the real needs of families.

This analysis agrees with the systematic review [18] results which state that programme duration, delivery mode, and location should be determined based on existing resources and systems, community needs, population risk profiles, and cultural context.

In summary, the present scoping review gives important clues in structuring parenting programmes related to the content, means to be used, and duration. Several models of structure may be used, but the literature is clear that these decisions affect the involvement and motivation of parents’ adhesion to the programme.

## 5. Conclusions

This study provides an overview of parenting programmes, and the results guide several recommendations for the future. 

The results of this article may help public health professionals to plan evidence-based parenting programmes with better outcomes for families, children, and society. These results will guide professionals in constructing more sustainable and effective parental programmes.

Considering the various benefits, such as increased parent competence, self-efficacy, lower parental stress, lower depression symptoms, and child behaviour problems, implementing parental programmes is an added value for parents, children, and society. However, professionals should be aware of the barriers identified in this study (related to distance strategies and individual needs) and carefully adapt any programme to individual, group or community needs.

Parenting programmes are important ways to contribute to a healthy, sustainable, and resilient society. They benefit parents by reducing signs of stress and depression and increasing their self-efficacy and social behaviour, which leads to positive changes in childcare. In addition, they bring benefits to the parent–child relationship by relieving social pressure from parents, allowing a focus on the relationship with the child as an individual. Finally, parenting programmes also positively affect society through knowledge sharing, networking, and support so that children can grow up in healthy environments and maximise their full potential.

## Figures and Tables

**Table 1:** Search terms identified in all databases included.

Database	Search Terms	Limiters
CINAHL Plus with Full Text (via EBSCOhost Research Databases)	(MM “Parenting Education”) (MM “Parenting Education”) AND (MM “Program Evaluation”) parent* AND program	Subject Age: infant, newborn: birth to 1 month old; all children Language: English, Portuguese
MEDLINE with Full Text (via EBSCOhost Research Databases)	(MM “Education, Nonprofessional”) AND (MM “Parenting”) (MM “Program Evaluation”) AND (MM “Education, Nonprofessional”) AND (MM “Parenting”) parent* AND program	
PubMed	(“Education, Nonprofessional” [Mesh]) AND “Parenting” [Mesh] ((“Education, Nonprofessional” [Mesh]) AND “Parenting” [Mesh]) AND “Program Evaluation” [Mesh] parent* AND program	Subject Age: all children Language: English, Portuguese
OpenGrey.eu; ProQuest; RCAAP; Google Scholar	parent* AND program Programa parental (parental programme, in Portuguese)	Language: English, Portuguese

**Table 2:** Characteristics of the included studies.

Study	Reference, Country	Population	Context	Concept	Methodology	Outcomes
1	[27], Australia	Parents who were primary caregivers of an infant or toddler aged under two years old	Community Family-organisations such as libraries and playgroups in Perth, Western Australia	Respectful approach intervention	Quasi-experimental mixed-method study	Benefits: Increase in parent competence and refocus on relationship with children; Reduce stress, depression; the sense of performance pressure.
2	[28], USA	English-speaking mothers with infants 3–12 months of age	Community The rural area of the Pacific Northwest	New brief temperament guidance programme for parents of infants	Mixed method study	Benefits: Increase knowledge of temperament and related concepts; representations of children’s attributes; sensitive interactions with their children.
3	[29], Australia	Parents of children aged 0–5 years	Community Playgroup Queensland (PGQ), a not-for-profit organisation	Community playgroups	Qualitative study	Facilitators: Practical strategies to apply the knowledge; Peer support group. Barriers: Tiredness or lack of time to participate.
4	[30], Australia and USA	155 parents (M = 33 years, SD = 7.5) with a 2- to 12-year-old child	Community	Triple P Online Community Programme	Mixed method study	Facilitators: Programme’s reward system; Social network. Barriers: Time; Technical problems; Content not adjusted to the child’s age.
5	[31], Spain	22 groups of 10–14 parents with a child 2–12 years old. 216 participants who completed the intervention	Community health strategy “Health in the Neighbourhoods”	Parenting Skills Programme for families (PSP)	Mixed method study: Quasi-experimental study design with pre (T0), post (T1), a follow-up (T2), and no control group, complemented by a qualitative study	Benefits: Improve parenting skills, children’s behaviours, parental stress, and social support immediately and six months after participation.
6	[32], USA	99 mothers	Community	Mom Power Programme	Mixed method study	Facilitators: Motivational interview.
7	[33], Australia	Parents of infants aged 6–12 months and parents of toddlers aged 12–36 months	Community-based parenting programme	Programme and community contextual factors on parent engagement	Two parallel randomised controlled trial	Facilitator factors: A structured curriculum; Home coaching.
8	[34], China	819 infants and their caregivers in 50 rural villages in north-western China	Community	Community-based ECD programme	Quantitative study	Facilitator factor: Social interactions. Barrier: Geographic distance.
9	[35], Australia	133 new mothers were referred by their birthing hospital for their initial postnatal health check by nurses	Community	Online group–based nurse-led intervention programme	Block randomised controlled trial.	Benefits: Helpful and user-friendly.
10	[36], USA	Two hundred thirteen primary caregivers of children ages 0–4 participated.	Community	Active Parenting First Five Years (FFY) programme	An inclusive randomised controlled trial	Benefits: Increase: responsive parenting, developmental knowledge, parent self-efficacy, mindfulness. Decrease: parenting stress.
11	[37], China	44 first-time primiparous women 18 years old or above were admitted to maternity wards of two public tertiary hospitals in China	Community	Internet-based support programme	Multicentre, single-blinded, randomised controlled trial.	Benefits: Increase: maternal self-efficacy and social support; Decrease postpartum depression symptoms.
12	[38], Canada	Four PDEP facilitators and seven parents (one male and six female) completed the programme	Community-primary prevention programme	Positive Discipline in Everyday Parenting (PDEP)	Qualitative study	Benefits: Created learn new ways of thinking about parenting; Contributed to changes in the parenting approach. Facilitator factors: Coherence and continuity among programme modules; Concrete examples to demonstrate concepts; Supportive facilitators and fellow group members; Childcare provision.
13	[35], Denmark	112 families with new-borns	Community	Incredible Years Parents and Babies (IYPB) programme	A two-arm parallel randomised controlled trial	No effects were found.
14	[39], China	149 parent–child dyads	Community	Parent and Child Enhancement (PACE) Programme	Randomised controlled trial	Benefits: Increase: child preschool concepts; Decrease: child behaviour problems and parental stress.

## Data Availability

Dataset is available upon request to the authors.
